# Calibration of Visible Light Positioning Systems with a Mobile Robot

**DOI:** 10.3390/s21072394

**Published:** 2021-03-30

**Authors:** Robin Amsters, Eric Demeester, Nobby Stevens, Peter Slaets

**Affiliations:** 1 Department of Mechanical Engineering, KU Leuven, 3000 Leuven, Belgium; Eric.demeester@kuleuven.be (E.D.); peter.slaets@kuleuven.be (P.S.); 2 Department of Electrical Engineering, KU Leuven, 3000 Leuven, Belgium; nobby.stevens@kuleuven.be

**Keywords:** indoor positioning, visible light positioning, sensor fusion, mobile robot, calibration

## Abstract

Most indoor positioning systems require calibration before use. Fingerprinting requires the construction of a signal strength map, while ranging systems need the coordinates of the beacons. Calibration approaches exist for positioning systems that use Wi-Fi, radio frequency identification or ultrawideband. However, few examples are available for the calibration of visible light positioning systems. Most works focused on obtaining the channel model parameters or performed a calibration based on known receiver locations. In this paper, we describe an improved procedure that uses a mobile robot for data collection and is able to obtain a map of the environment with the beacon locations and their identities. Compared to previous work, the error is almost halved. Additionally, this approach does not require prior knowledge of the number of light sources or the receiver location. We demonstrate that the system performs well under a wide range of lighting conditions and investigate the influence of parameters such as the robot trajectory, camera resolution and field of view. Finally, we also close the loop between calibration and positioning and show that our approach has similar or better accuracy than manual calibration.

## 1. Introduction

Since their introduction, Global Navigation Satellite Systems (GNSS) are the enabling technology for applications such as navigation, autonomous vehicles and emergency services. While GNSS can provide worldwide coverage and require only a receiver to use, they are typically not useful for indoor spaces. On one hand, building walls significantly reduce the signal strength, often making positioning impossible or reducing the accuracy [1]. However, even the nominal accuracy of GNSS (around 5 m [2]) is insufficient for indoor positioning, where an error of a couple of meters can mean that the user is located in one of several rooms. In order to provide indoor location, researchers have developed many Indoor Positioning Systems (IPS), yet a single standard like GNSS was not achieved. Indoor environments come in many different varieties and can favor different positioning technologies. Current systems are often based on Wi-Fi in order to reduce infrastructure cost; however, their accuracy is limited to a couple of meters [3]. Other technologies such as Ultra-WideBand (UWB) [4] and ultrasound [5] can provide much higher accuracy (centimeters), at the cost of additional specialized infrastructure.

With the introduction of solid-state lighting, a new type of indoor positioning has emerged. In Visible Light Positioning (VLP), light intensities are modulated at speeds imperceptible to the human eye, which allows for a one way transmission from transmitter to receiver. Similar to other positioning systems, the Received Signal Strength (RSS) or signal travel time can be used to determine the receiver location. Due to the local character of light, the influence of multipath is significantly reduced, resulting in an accuracy that can be as low as a couple of centimeters [6]. Existing lighting infrastructure can also be reused for positioning, thereby reducing the overall system cost significantly. These advantages led to an increasing research interest in recent years. However, the installation of new VLP systems remains an important issue. The majority of indoor positioning systems require some form of calibration. For example, systems that use range measurements to determine the receiver position via triangulation assume the locations of all transmitters to be known. Manually measuring transmitter locations can be a cumbersome process, as the transmitters are often mounted on ceilings and walls [7]. Fingerprinting systems also require a calibration procedure, in order to build an RSS map that can later be used for positioning. These site surveys can be lengthy and labor-intensive processes. Additionally, this RSS map may have to be updated when changes occur in the environment.

Several calibration procedures have been proposed for IPS using technologies such as UWB [8], Wi-Fi [9,10] and Radio Frequency Identification (RFID) [11,12]. In the field of visible light positioning, little literature is available on this subject. Our previous work [13] proposed a proof-of-concept calibration procedure with a mobile robot. In that procedure, the total number of lights needed to be determined manually [13]. Counting the number of transmitters is significantly less time-consuming compared to manually measuring the positions. However, it is still a tedious process that is prone to errors. In this work, we therefore introduce an improved calibration algorithm. Detected light sources are filtered based on their measured coordinates, as well as their place in the frequency spectrum. Using this new procedure, the number of light sources is no longer required. Additionally, accuracy is significantly improved. As [13] is a proof-of-concept, much remains unknown about the robustness of the approach. For example, which parameters have an effect on the accuracy of the procedure? To find out, we investigate the impact of a variety of factors on the calibration procedure. Finally, the goal of a calibration procedure is to prepare the system for positioning. The relation between calibration errors and positioning errors may be complex. In order to determine whether our system has satisfactory performance, we use the calibrated parameters for positioning. Following this approach, we close the loop between calibration and positioning and enable high-performance systems that are easy to deploy.

Our main contributions can therefore be summarized as follows:

- Improved calibration procedure with nearly double the accuracy compared to previous work [13].
- Extensive parameter study that investigates the influence of the transmitter waveform, lighting conditions, robot trajectory, camera resolution, Field Of View (FOV) and transmitter–receiver distance.
- The calibrated parameters are used for positioning. The accuracy of the resulting location data are evaluated independently from the calibration.

The rest of this paper is structured as follows: Section 2 describes related work, and Section 3 introduces the materials and methods used in this paper. Experimental results are presented in Section 4 and discussed in Section 5. Finally, a conclusion is drawn in Section 6.

## 2. Related Work

Table 1 provides an overview of calibration procedures proposed for different types of indoor positioning systems. In Table 1, “positioning technology” refers to the technology that is actually used for positioning (after the calibration has completed). During the calibration itself, other signals such as RGB-D cameras [10] or PDR [14] may be used. The following section will describe the broad categories of calibration methods in more detail.

Fingerprinting-based IPS operate in two stages. In the first (offline) stage, a signal strength map is constructed. RSS values are measured at known locations throughout the entire space. It is possible to record just one type of signal (e.g., Wi-Fi). However, accuracy is generally improved by including multiple sources of information (e.g., magnetic field, Bluetooth, etc.) [38]. Signals already present in the environment are often used, in order to avoid the need for additional infrastructure. In the second (online) stage, the receiver location is unknown and one or more RSS values are measured. By matching the current signal fingerprint to the database, the receiver position is recovered. Contrary to triangulation-based IPS, fingerprinting approaches do not require transmitter coordinates. To ensure positioning accuracy, it is however important that the signal strength map is accurate. The map may also have to be updated periodically, if changes to the environment are made.

We distinguish four methods to construct the signal strength map:

- Manual site survey
- Robot site survey
- SignalSLAM
- Crowdsourcing

In manual site surveys, a trained expert records signal fingerprints at known locations. The entire space needs to be visited by the surveyor and as mentioned before, this process may have to be repeated. Manual site survey is time-consuming and labor-intensive and is thus not always practical in large indoor spaces [35]. The use of mobile robots has therefore been proposed to simplify this task. Mobile robots have been used to collect fingerprints for RFID [11,12] and Wi-Fi systems [10]. Some authors have even proposed algorithms that enable a robot to collect data without human intervention [9,15]. These navigation algorithms were relatively simple and did not follow the optimal trajectory (in terms of accuracy or time required), but they did succeed in covering the space eventually.

The goal of Simultaneous Localization And Mapping (SLAM) is to reconstruct a map of the environment, while simultaneously estimating the trajectory of the observer relative to that map. Solutions to the SLAM problem are most commonly based on Bayesian filtering [39]. SLAM algorithms have mainly been used for robotics applications, as earlier implementations required expensive sensors such as laser scanners (LIDAR) or depth cameras [40]. Recently, researchers started using sensors embedded in conventional smartphones to construct signal strength maps. This approach is sometimes also referred to as signalSLAM. Pedestrian Dead Reckoning (PDR) is often used to obtain a rough estimate of the user’s trajectory, and drift is corrected by using absolute location fixes (for example, from GNSS signals or near-field communication tags) [16]. Alternatively, other signals of opportunity such as Wi-Fi, magnetic field or even ambient light [35] can be used to compensate PDR drift. When using signalSLAM to calibrate fingerprinting IPS, the main goal is to reconstruct the trajectory of the user and to add the measured signal strengths to the map based on that trajectory. Recent approaches tend to use a modified version of graphSLAM [36]. The main challenge with graph-based signalSLAM is the reduction of false positives when performing loop closures [14,16,17].

SignalSLAM calibration still requires surveyor to visit the entire indoor space. It is more efficient compared to manual site survey, as the surveyor can walk around continuously. In manual calibration, the surveyor has to stop and record his or her location periodically. Crowdsourcing approaches attempt to improve efficiency even further by removing the dedicated surveyor entirely. Initially, users can go about their regular tasks, while the systems collect both inertial and signal strength data from their smartphones in the background. As more data are collected, these systems obtain a more complete picture of the indoor environment, and position accuracy increases. In contrast to single site surveys, the map can continuously be updated. Crowdsourcing presents a number of interesting advantages, yet some challenges still remain. Kim et al. [18] assumed the initial location of the user was known and suggested it can be obtained from GNSS when the user enters the building. In contrast, the system described in [19] did not require the initial position, stride length or phone placement. Instead, a map of the environment was used to impose constraints that can filter improbable locations. The obtained trajectories were optimized through backpropagation, and Wi-Fi signal strength was added to the map based on the optimized path. In the work of Wang et al. [20], seed landmarks were extracted from the floor plan (e.g., doors), which can be used to obtain global observations. Additional landmarks were learned as more data entered the system. Yang et al. [21] first transformed the map into a stress-free floor plan, which is a high dimensional space in which the distance between two points reflects their walking distance (taking constraints such as walls into account). The similarity between the stress-free floor plan and the fingerprint space was used to label RSS signatures with their real locations. Crowdsourcing-based calibration does require users to give up their personal data, which may be an important barrier to some. Moreover, the approaches discussed above often required a floor plan, which may not always be available. Finally, the accuracy of both signalSLAM and crowdsourcing is typically low (in the range of several meters). Due to the relatively low quality input data (PDR and radio frequency signals), it is challenging to obtain robust and accurate systems with signalSLAM or crowdsourcing.

The calibration methods discussed so far are only applicable to fingerprinting-based IPS. Another category of positioning systems obtains the receiver position based on ranging. The travel time of a signal or the signal strength are used to determine the distance between transmitter and receiver. From the measured distances, the receiver position can then be obtained via triangulation. These types of IPS require accurate knowledge of the transmitter locations. Depending on the positioning technology used, additional parameters may also be required. For example, UWB systems often correct the bias on the distance measurements [8]. VLP systems based on RSS sometimes calibrate the gain [30] or Lambertian emission order [33]. For the calibration of range-based systems, we can again distinguish a few possible methods:

- Manual measurements
- Known locations of receiver(s) and/or transmitters
- Interbeacon ranging (autocalibration)
- Network optimization

Similar to fingerprinting systems, range-based IPS can be calibrated manually. In this case, the transmitter locations would be measured relative to some reference with rulers or laser-based measurement devices. While measuring the transmitter locations manually requires less work than performing a manual site survey for fingerprinting, it is still a tedious process. Transmitters are often mounted on the ceiling, which can make the process somewhat inconvenient. Ranging systems can also be calibrated based on known receiver locations, which may be easier to obtain than the transmitter coordinates [22,23,24]. However, ground truth measurements of the receiver locations are still required, which often requires an additional positioning system. Moreover, errors on the receiver location while calibrating will subsequently lead to errors on the transmitter locations. If sufficient transmitter positions are known, the others can be extrapolated without extra measurements [25].

Some IPS can use the same ranging techniques that enable receiver positioning to obtain the distance between transmitters, from which the transmitter locations can also be obtained [27,28]. These interbeacon ranging techniques (sometimes also referred to as autocalibration) do assume that beacons can communicate with each other. Additionally, the transmitters must be placed sufficiently close together such that they are within measurement range of each other, which may disqualify them from positioning technologies such as Bluetooth.

Finally, range-based IPS can also be calibrated based on a set of transmitter–receiver distances. If the quantity of data is large enough, no receiver or transmitter locations are required; a set of range measurements is sufficient. Calibration can then be formulated as an optimization problem that minimizes the residual of the trilateration equations [7,8,26,27,29]. Results from these approaches are not always unique, for example, in the case of rotational symmetry. Additionally, accuracy of the solution can be heavily dependent on the initial conditions [29].

Both ranging and fingerprinting can be used for VLP. Ranging is generally more accurate and robust. However, as the transmitters are lights that also illuminate the space, they are generally mounted on the ceiling and are pointing downwards. Therefore, transmitters likely do not have a line of sight (LOS) to each other. Even when VLP transmitters are within range of each other, they lack the necessary hardware for receiving signals. Therefore, autocalibration methods cannot be used by most conventional VLP systems. In fact, VLP calibration in general is not yet explored in depth. Rodríguez-Navarro et al. [30] proposed a method for calibrating the electrical parameters of a VLP amplification circuit. They performed an extensive parameter study and found that manufacturing tolerances on the resistors and capacitors contributed most to positioning errors due to incorrect calibration. By performing multiple intensity measurements at known locations, a system of equations can be constructed. The solution that minimizes the error provides the optimal calibration of the receiver parameters. In [31,32], calibration of transmitter coordinates based on known receiver locations was proposed. Similarly, Ref [33,37] were able to calibrate the channel model based on known receiver locations. However, these works either did not indicate how the receiver position should be obtained [31,32,33] or used an additional positioning system to obtain it [37]. Note that not all VLP systems require a calibrated channel model. Camera-based implementations such as [41] only detect the relative position of the light to the camera center, while photodiode-based systems use the signal strength to obtain the transmitter–receiver distance. Camera-based VLP systems therefore only need the location of each transmitter. However, the channel model of VLP is relatively well known; therefore, model-based fingerprinting is sometimes also possible given the transmitter locations [34].

In this work, we will focus on the calibration of light source locations and identifiers without prior knowledge or additional positioning systems, of which there are few examples. Liang and Liu [35] crowdsourced the construction of a signal strength map of opportunistic signals. Similar to [14,16], user trajectories were obtained with the help of a modified graphSLAM algorithm. Contrary to similar works, they also mapped the location of light sources and used them as landmarks in the positioning stage. However, as the lights were not modulated, their identity cannot be uniquely determined, resulting in a relatively low positioning accuracy (several meters) [35]. Additionally, unmodulated light sources are not easily distinguished from sunlight, as both increase the ambient lighting. In contrast, Yue et al. [36] did use modulated Light Emitting Diodes (LED). A modified version of graphSLAM was again used to construct the signal strength database. Absolute location fixes were obtained by detecting doors with changes in light intensity and magnetic field strength. Following calibration, positioning was performed by fusing PDR with fingerprint observations via a Kalman filter. Positioning accuracy after calibration was about 0.8 m on average, which is an improvement of approximately 70% over Wi-Fi-based fingerprinting under the same conditions. However, in rare occasions the positioning error can exceed 2 m.

## 3. Materials and Methods

**Figure 1:**
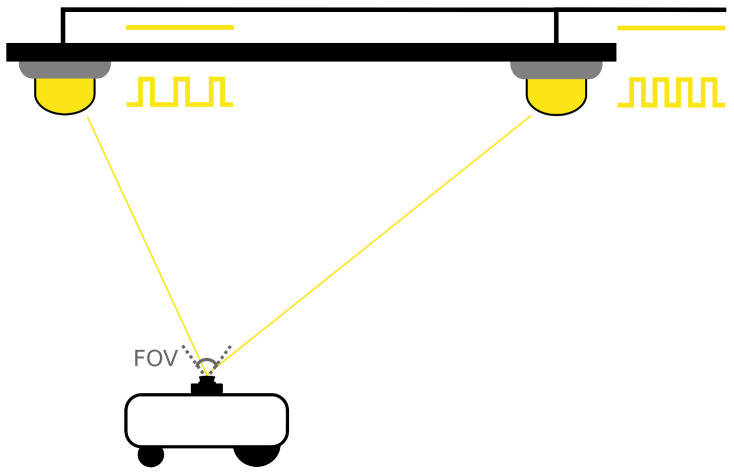
Model of the proposed Visible Light Positioning (VLP) system.

In our proposed system, specific hardware was placed between the power lines and the lights, which modulated the intensity of each LED at a unique frequency (see Figure 1). Contrary to VLC, no data was transmitted. Instead, we used the modulated lights as a landmark. Detection and identification of the light itself is not the focus of this paper but was explained in our previous work [13]. The main variables of interest were the identity (i.e., frequency) and the coordinates in the camera frame of the light source. If the position of each light is known beforehand, this information can be used to obtain the receiver location. However, in this work, we will focus on the calibration itself. We chose frequency division for its easy implementation (see Section 4.2.1), but another modulation technique could also be used. So long as the light sources can be detected (within the field of view) and identified, the calibration procedure remains applicable.

### 3.1. Experimental Setup

**Figure 2:**
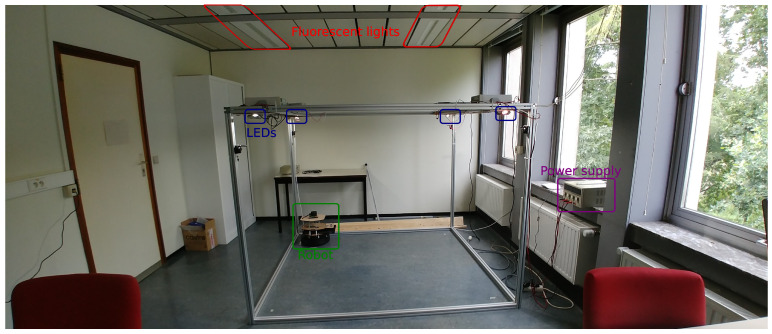
Experimental setup used in this paper, which was also used in our previous work [13,41].

In order to evaluate the proposed calibration procedure, we used the same experimental setup as our previous works [13,41] (see Figure 2). Four VLP transmitters were mounted at a height of approximately 1.5 m. Light intensity of every transmitter was modulated by a Metal-Oxide Semiconductor Field-Effect Transistor (MOSFET) that was connected to a signal generator. Every LED had a unique frequency between approximately 1.5 kHz and 5 kHz, so that both low and high frequency modulation can be evaluated. The complete methodology used to obtain suitable transmitter frequencies was detailed in [13]. Table 2 lists the selected modulation frequencies, along with the other main hardware specifications.

**Figure 3:**
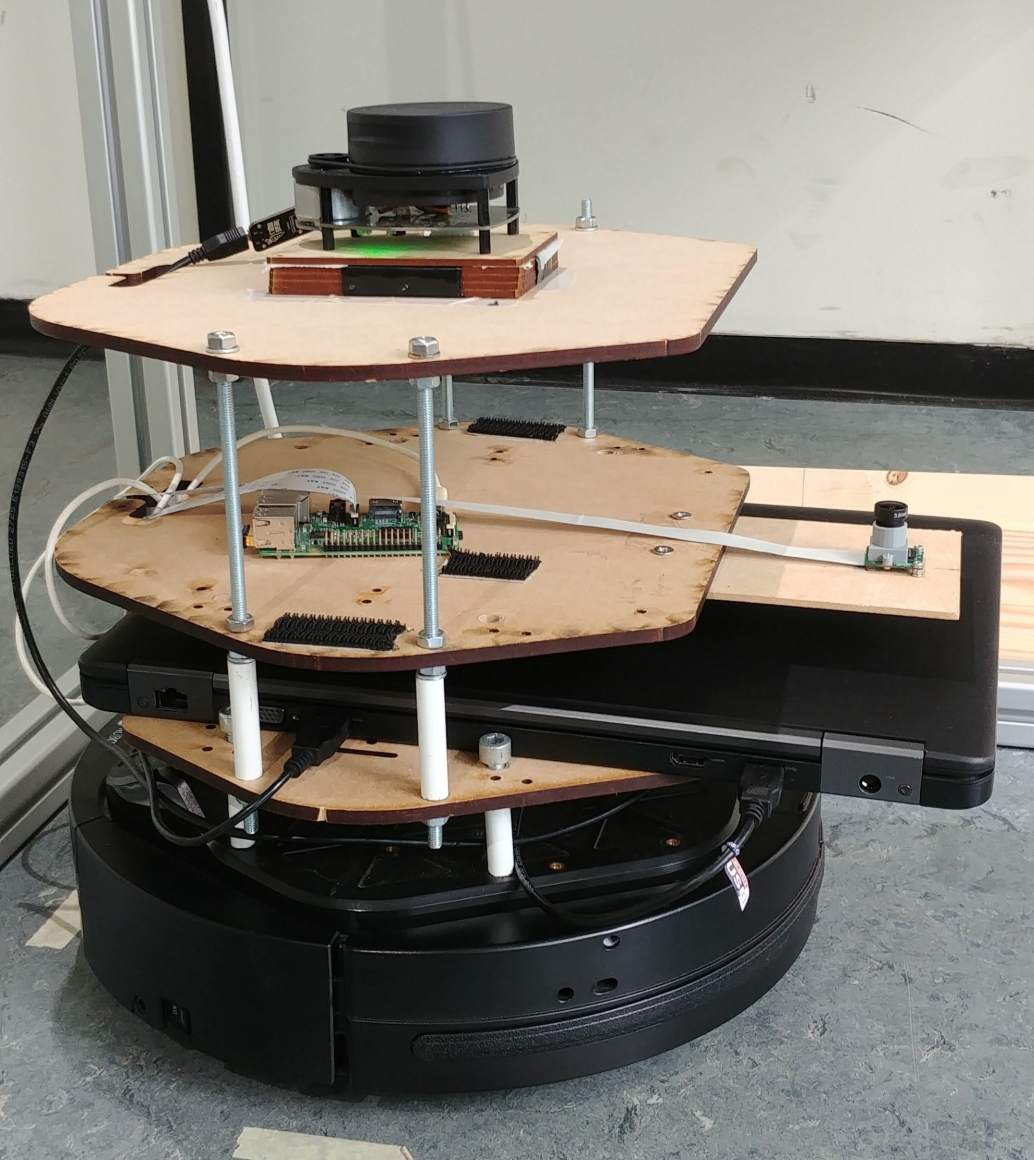
Mobile robot with custom sensor platform used in this paper, which was also used in our previous work [13,41].

A mobile robot was equipped with a custom sensor platform that contained a laser scanner and a camera (see Figure 3). Two different cameras will be investigated in this paper. Initially, we used the OpenMV M7 camera during experiments, as the low resolution allowed us to more quickly process the images and therefore speed up development. Later experiments used the Raspberry Pi (RPi) camera module. Similar to the OpenMV M7, the RPi camera allows a high flexibility over settings such as the exposure time. However, the RPi camera has a much greater resolution compared to the OpenMV camera. Section 4 will investigate whether this resolution improves accuracy. The sensor platform also contained a laptop, which recorded all data so that calibration could be performed offline. When the RPi camera module was used, a Raspberry Pi single-board computer recorded the images separately from the laptop, as the RPi camera does not have a Universal Serial Bus (USB) interface. The cost of the main components of the experimental setup is detailed in Appendix A. The robot platform was driven by a human operator via a remote control.

### 3.2. Calibration Procedure

**Figure 4:**
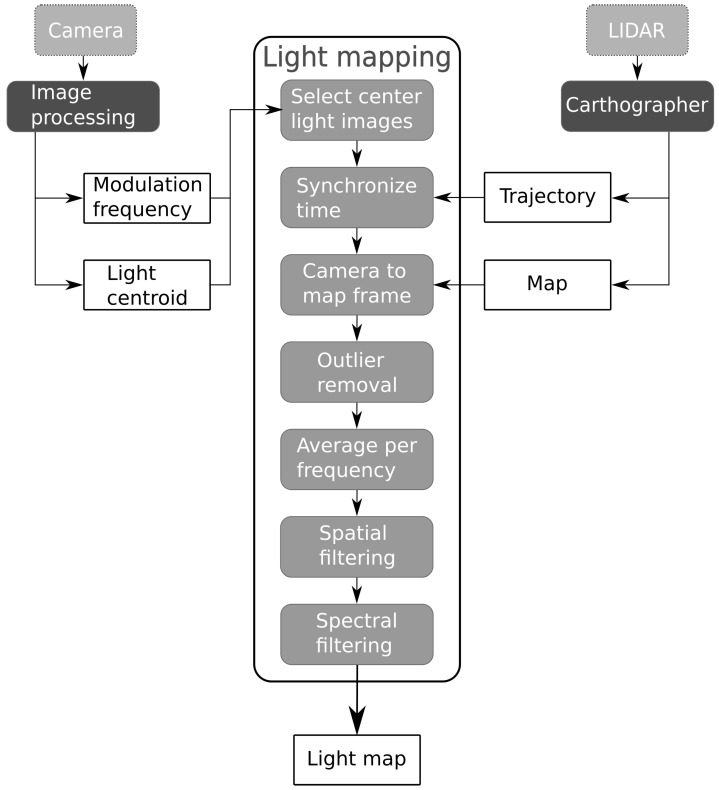
Calibration procedure overview.

Figure 4 provides a graphical overview of the calibration procedure. The parameters used to obtain the results in Section 4 are listed in Table 3. The initial steps of the improved procedure were still the same as in [13]. Due to the rolling shutter of our Complementary Metal-Oxide-Semiconductor (CMOS) camera, modulated light sources are visible as stripe patterns in the images. The width of the stripes is proportional to the transmitter frequency [42]. The complete image processing pipeline is detailed in our previous work [13] and returns the frequency and pixel coordinates of the lights as output. The effects of lens distortion are largest at the edges in a picture. Therefore, only the images where a light was detected close to the image center were processed further. Measurements of the laser scanner were used to reconstruct the followed trajectory and a map of the environment by using the Google Cartographer SLAM algorithm [43]. By combining the trajectory of the robot with the detected light sources, we obtained a map of the environment with the light source locations relative to the map frame.

**Figure 5:**
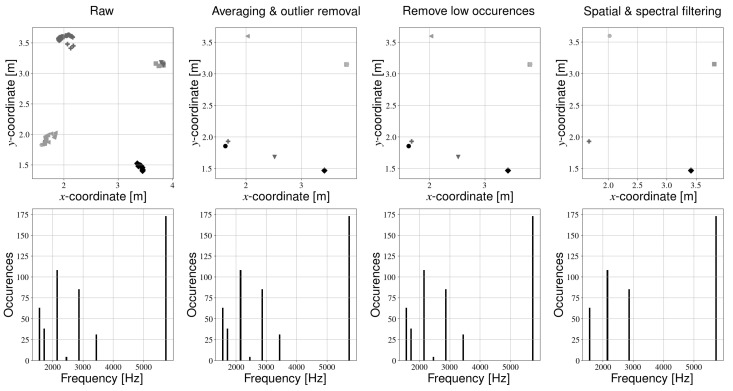
Filtering pipeline. The top row always indicates a light map, and a unique marker is used to indicate different frequencies. The bottom row shows the frequency spectrum corresponding to a certain processing step. From left to right: (1) raw output of the light mapping step, (2) light map after averaging coordinates of unique frequencies and removing outliers (3) light map after removing spurious observations (4) final results of spatial and spectral filtering.

At this point, we have obtained the world coordinates of detected light sources, in addition to their frequencies. Unfortunately, the same LED is occasionally labeled with different frequencies, depending on the image. The Canny edge detection step may calculate the stripe pattern to be one pixel larger or smaller than the actual value, resulting in a small spread in the frequency spectrum (see Figure 5). Additionally, the detected world coordinates of the light sources may also not be a single point, due to noise on the centroid detection and robot localization. The light may therefore appear as a cluster of coordinates in the light map (see Figure 5). Previously, we solved this problem by averaging the world coordinates per detected frequency. Then, the frequencies which were detected most often were kept, depending on the number of light sources. For example, in our experimental setup four lights are present; therefore, the calibration procedure selected the four frequencies that were detected most often, and the rest were discarded. This approach has the disadvantage that one first needs to know how many light sources are installed in the environment, and therefore some manual measurements may still be required.

We now propose a different method, whereby we filter the light sources based on their physical coordinates and the frequency spectrum. The intermediate results of the processing steps can be seen in Figure 5. First, outliers are removed from the detected coordinates per frequency. Outliers are defined as detected positions that have a distance of more than 2 standard deviations from the average position. Then, the coordinates of the remaining light sources are averaged per frequency. Light sources that were only observed a few times are removed. Next, light sources that are close to each other are combined. Filtering is first performed based on the coordinates of the light sources. Whenever the distance between two transmitters is below a certain threshold (Table 3), the LED with the lowest number of observations is removed. In case both lights have an equal amount of detections, their detected frequencies and positions are averaged. We call this step “spatial filtering”. Finally, we combine light sources of approximately equal frequency. Whenever the distance in the frequency spectrum of two light sources is below a certain threshold (Table 3), the frequency with the largest number of observations is kept and the other is removed. Similar to the spatial filtering step, we average the frequencies and positions of light sources with an equal amount of detections. We call this final step “spectral filtering”. The result of these additional processing steps is a light map with the correct number of transmitters. The number of light sources therefore no longer needs to be known beforehand. Instead, one only needs to know (approximately) how far lights are minimally spaced apart, which is much easier to obtain.

### 3.3. Data Processing

The result of the calibration procedure is a map of the environment that includes transmitter locations and their frequencies. Evaluating the accuracy of frequency detection is relatively straightforward and was performed by comparing the frequency applied by the signal generator to the frequency determined by the calibrating procedure. Evaluating the location of the light sources is more complex. Ideally, we could simply compare the coordinates determined by the calibration procedure to the coordinates in the physical setup. However, the calibration procedure produces coordinates relative to the map, which is not necessarily the same coordinate frame as the experimental setup, and obtaining the transformation between these frames is challenging. However, we can still compare the relative placement of the light sources. The distance between different light sources is irrespective of the coordinate frame. Therefore, in order to obtain the transmitter position accuracy, we subtracted the distance in the physical setup from the distance obtained in the light map. The distance error between two lights was therefore calculated by:

(1) $$\varepsilon_{r,ij}=\left|d_{meas,ij}-d_{est,ij}\right|=\left|d_{meas,ij}-\sqrt{{\left(x_{est,i}-x_{est,j}\right)}^{2}+{\left(y_{est,i}-y_{est,j}\right)}^{2}}\right|$$

where:

- $\varepsilon_{r,ij}$ is the error on the distance between lights *i* and *j*
- $d_{meas,ij}$ is the manually measured distance between lights *i* and *j*
- $d_{est,ij}$ is the distance between lights *i* and *j* as estimated by the calibration procedure.
- $x_{est,i}$ and $y_{est,i}$ are the estimated Cartesian coordinates of light source *i*
- $x_{est,j}$ and $y_{est,j}$ are the estimated Cartesian coordinates of light source *j*

In Section 4, we will investigate the performance of the system under a range of conditions. Unless explicitly mentioned otherwise, three experiments are conducted for every condition and the results from all three experiments are combined before further processing (for example, in order to obtain the cumulative error distribution).

## 4. Results

### 4.1. Baseline Results

**Figure 6:**
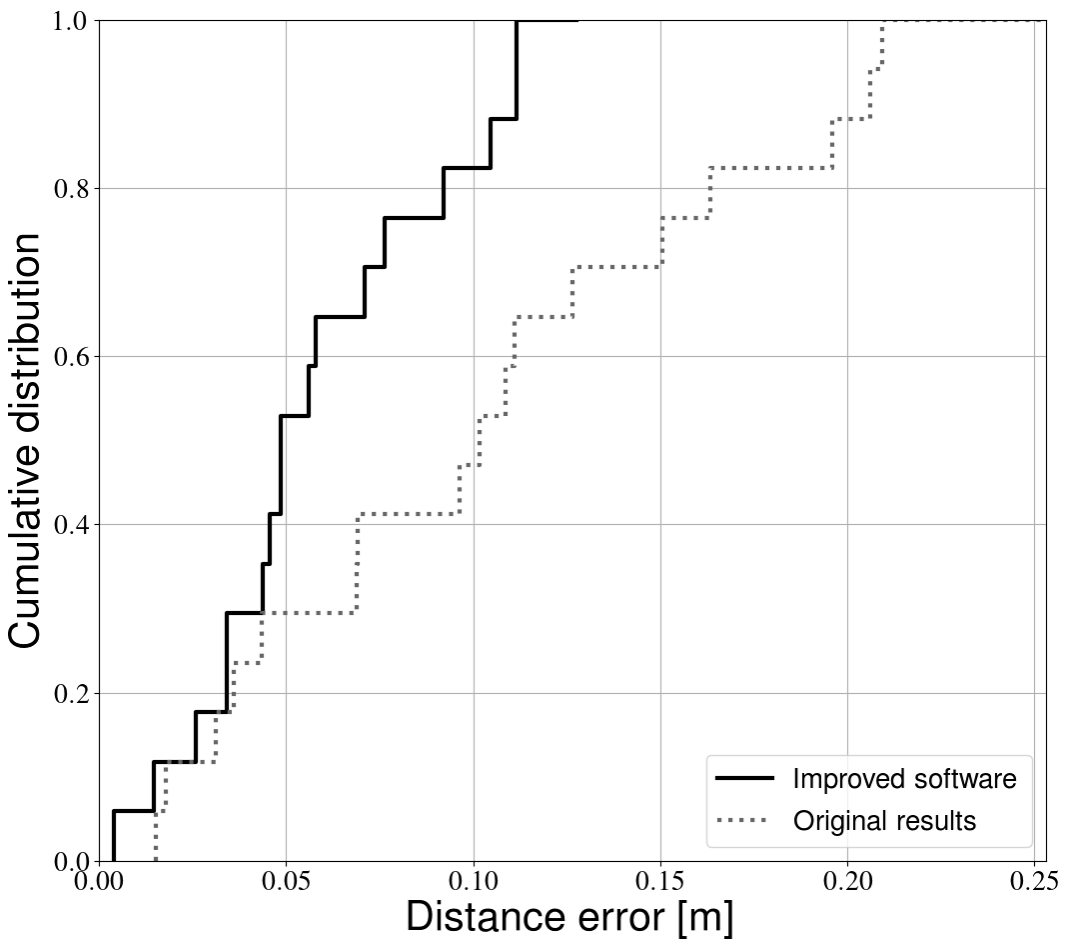
Cumulative error distribution of this paper compared with our previous work [13].

Figure 6 compares the calibration results obtained in [13] with the method proposed in this paper. It is clear that the additional filtering steps significantly improve the calibration accuracy. On average, light sources can now be detected with an accuracy of approximately 6 cm, compared to 11 cm in [13]. Larger improvements are also visible in the higher percentiles of the distribution. More than 80% of light sources can be positioned with an accuracy of 10 cm, compared to 20 cm in [13]. Note that the same experimental data were used to obtain both error distributions; the difference is therefore purely due to improvements in data processing.

While this new method can localize the LEDs more accurately, the results of the frequency detection remain unchanged. As was the case in [13], it is challenging to calibrate the high frequency transmitter. As the frequency increases, the width of the stripes decreases, and a detection error of a few pixels results in a large frequency error (error of several hundred Hz). The lower and medium frequency sources can, however, be identified with relatively high accuracy (error of maximum 130 Hz).

### 4.2. Parameter Study

Using a mobile robot is a completely new approach to the calibration of VLP systems. As with any new technique, much is currently unknown about the effects of certain parameters on the calibration results. In the following sections, we will therefore investigate the influence of a number of factors on the calibration procedure. One parameter will be changed at a time, and unless otherwise specified, we will use the results from the previous section as a baseline to compare against. In doing so, we aim to create a better understanding of the strengths and limitations of the proposed approach.

#### 4.2.1. Transmitter Waveform

In visible light positioning, the light intensity of every LED is modulated in such a way that they are uniquely identifiable, even when multiple lights are in view at the same time. Generally, the lights continuously transmit a unique code or frequency [44]. The former is referred to as Code Division Multiple Access (CDMA), the latter as Frequency Division Multiple Access (FDMA). In this paper, we use FDMA as a multiple access technology. However, the calibration procedure can be adapted to support CDMA as well with relatively minor changes.

When using FDMA, both sine waves and square waves can be used as transmitter waveforms. A square wave is easier to generate and therefore the cost of the transmitters can be lower. Therefore, most VLP systems in literature use square waves. However, square waves have harmonics in the frequency spectrum. When selecting square wave frequencies, more care is needed to avoid interference. Photodiode-based VLP systems use the Fourier spectrum to separate the received signal into the components of each transmitter. Therefore, photodiode-based VLP systems are impacted most by harmonics. Camera-based VLP systems can use spatial multiplexing and are therefore less affected by this interference.

**Figure 7:**
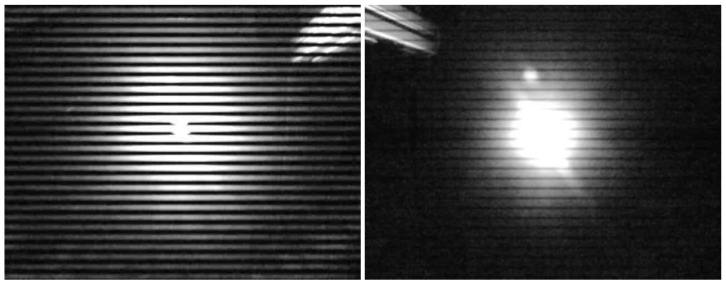
Comparison of 2 kHz square wave (**left**) and sine wave (**right**).

On the other hand, ideal sine waves have no harmonics, and therefore the available bandwidth can be used much more efficiently when photodiodes are used as receivers. The downside is that a sine wave is not as straightforward to generate with low-cost components. Additionally, the light intensity changes much more gradually with a sine wave, which makes frequency detection significantly more challenging (see Figure 7). In the following sections, we will therefore only use square waves, as our calibration procedure is not able to detect sine waves with sufficient accuracy.

#### 4.2.2. Robot Trajectory

**Figure 8:**
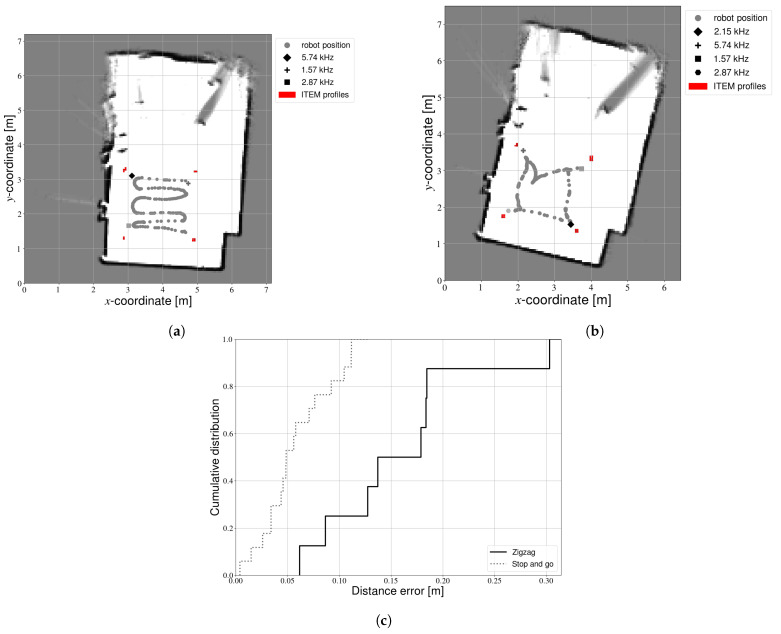
Calibration experimentswith different trajectories. (**a**) Zigzag trajectory. (**b**) Stop and go trajectory. (**c**) Cumulative error distribution.

The motion of the robot platform may influence calibration results. If the robot can stay in motion, calibration time will be reduced. On the other hand, we expect motion blur may negatively impact results. During experiments, the robot was driven manually via a remote control. Two different types of trajectories were tested. In the first trajectory, the robot was continuously in motion and passed by every light source while covering the experimental space in a zigzag pattern. Figure 8a shows an example light map constructed from data recorded during a zigzag experiment. Performance is quite poor in this example—one light source was not even mapped at all. Other zigzag experiments occasionally resulted in even fewer light sources. Moreover, Figure 8c shows that the positioning accuracy of the fixtures that were detected is quite low. We therefore also tested a second type of trajectory, whereby the robot drives towards each light source sequentially. Once the camera is directly below the LED, data were recorded for a few seconds, before continuing to the next light. We call this as a “stop and go” trajectory, Figure 8b shows a light map constructed based on data from such an experiment. The light sources are now placed closer to the ITEM profiles (see Figure 2). Additionally, Figure 8c shows that the relative placement is significantly more accurate.

Table 4 compares the duration of the two types of trajectories, based on the average of three experiments for each type. Contrary to our expectations, we can observe that performing the calibration with the stop and go trajectory does not take significantly more time. The zigzag is an exhaustive search, and therefore takes a long time. In contrast, the stop and go trajectory is not a continuous motion but only gathers the data that is really required. Processing time for the stop and go trajectory is slightly increased compared to the zigzag. Fewer images were rejected, as more light sources were detected close to the center for this type of experiment. Consequently, more images needed to be processed and the processing time increased. However, the majority of this time was actually spent on LIDAR mapping (approximately 80%). Therefore, an increase in image processing only had a small impact on the overall time required. Additionally, the difference is less than 1 s. As the calibration can be performed offline, this time delay does not present an obstacle.

Many other types of trajectories could be considered. Determining the optimal calibration trajectory is outside the scope of this paper. With these results, we can however conclude that the robot should briefly stop at each LED in order to obtain accurate results. Unless otherwise specified, results in the following sections are obtained with a stop and go trajectory.

#### 4.2.3. Lighting Conditions

Results from the previous section were all obtained under the same lighting conditions. It is well known that changing illumination levels can influence computer vision algorithms, and could thus negatively impact our proposed calibration procedure. In this section, we will therefore calibrate the experimental setup under a range of lighting conditions. More specifically, we distinguish 4 scenarios:

- Baseline: These lighting conditions applied to the results of the previous sections. More specifically, the shutters of the windows were closed, and no other light sources besides the LEDs were present.
- Other day: These experiments are conducted under the same circumstances as the baseline (closed shutters and no other light sources present) but on a different date approximately three months later. Baseline experiments took place in early spring when the sun sets earlier. In contrast, the “other day” experiments took place in summer, when the sky is clearer and the sun sets much later.
- Shutters open: During these experiments, the shutters of the windows were opened to allow sunlight to enter the room. These experiments also took place at the later date compared to the baseline.
- Fluorescent lights on: In addition to opening the shutters, the fluorescent lights are now also switched on. Fluorescent light is modulated and can in theory produce stripe patterns in our images. These experiments took place on the same day as the “Other day” and the “Shutters open” experiments.

**Figure 9:**
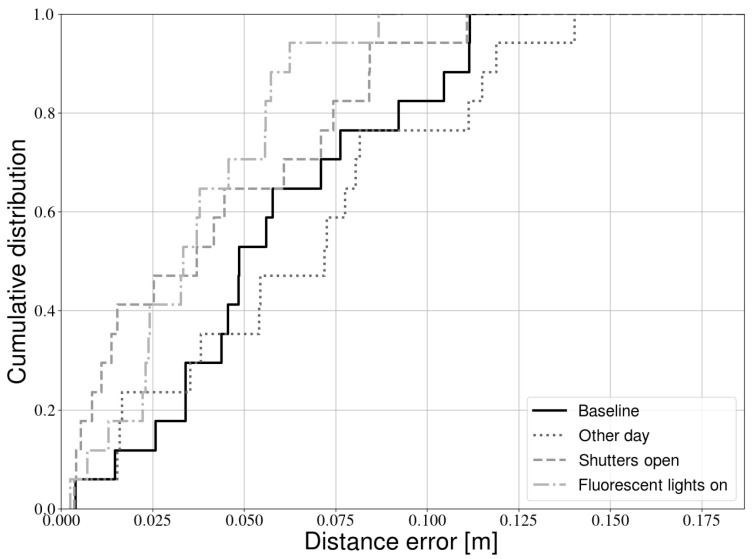
Influence of environmental conditions on calibration accuracy.

For every condition, three experiments were again conducted with the stop and go trajectory. For every experiment, we determined the error on the light source location as explained in Section 3. Figure 9 shows that all conditions have similar performance. The “other day” experiments are very similar to the baseline, indicating that the parameters (e.g., exposure time) are not overfit to a specific point in time. The difference between both error distributions generally is not larger than 2 cm. Whether or not the shutters are opened also does not seem to negatively impact calibration accuracy, as unmodulated light sources are easily ignored by our calibration procedure. Similarly, we can observe that switching on the fluorescent light has little impact. While fluorescent light is modulated, the frequency is too low compared to the LEDs. Therefore, the additional light sources are simply ignored.

The results of the frequency detection were very similar under different lighting conditions. In fact, they were identical with only one exception. During one “shutters open” experiment, one medium frequency light source had a slightly larger error compared to the other experiments. This phenomenon did not occur during the experiments with the fluorescent lights, even though the shutters were also opened in this case.

#### 4.2.4. Transmitter–Receiver Distance

**Figure 10:**
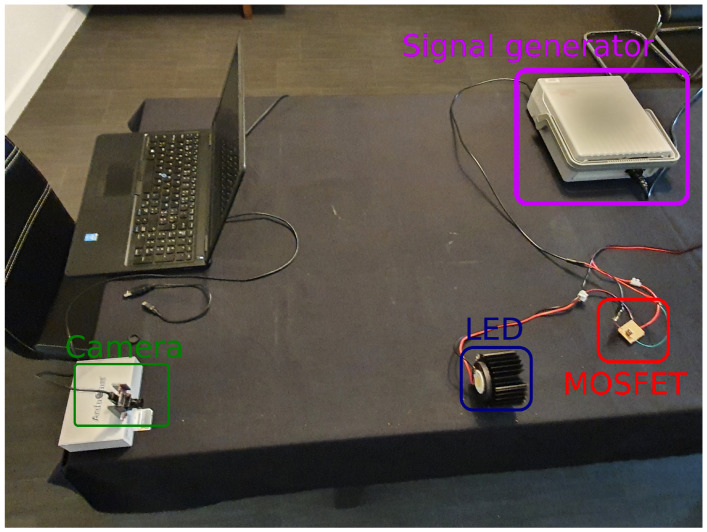
Experimental setup for transmitter–receiver distance experiments.

Experiments so far were performed with LEDs mounted at a height of approximately 1.5 m. This makes them more easily accessible and thereby makes prototyping and experimenting easier. As the distance between transmitter and receiver increases, the LEDs will take up a relatively smaller portion of the image. Consequently, fewer stripes will be visible, and it will be more challenging to determine the transmitter frequency. This section will characterize the influence of increasing this distance on the accuracy of frequency estimation. To that end, we place the camera and an LED on a table and ensure that their normal planes were parallel. This horizontal setup (Figure 10) was different from how the LEDs are normally installed. However, whether the lights are mounted horizontally or vertically made no difference for this experiment; only the relative distance is important. In contrast, placing the light on a table rather than on the ceiling allowed us to change the distance much more easily and also enabled us to test performance at larger distances.

**Figure 11:**
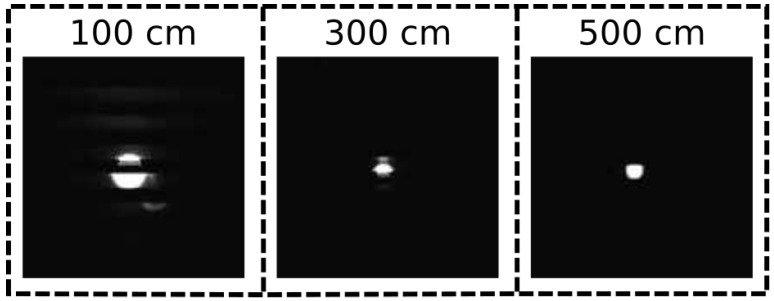
Example images taken at different transmitter–receiver distances. Images are cropped around the light source.

The distance between transmitter and receiver was increased from 1 m to 5 m. The light was modulated at a frequency of approximately 2 kHz. At every distance increment, images were recorded for approximately 30 s. In postprocessing, we determined the LED frequency using the process described in Section 3.2. Next, we calculated the difference between the true frequency (applied by the signal generator) and the frequency estimated by the calibration procedure. Table 5 shows the frequency estimation accuracy as a function of the transmitter–receiver distance. For short distances, the accuracy is approximately 95%, similar to Section 4.1. However, starting at a distance of 2 m, light sources can no longer be detected, hence the accuracy is 0%. The cause for this problem can be found by comparing images captured at different distances (Figure 11). At a distance of 1 m, several horizontal stripes are visible, from which the transmitter frequency can be calculated. At a distance of 3 m, only 1 stripe is visible, and the transmitter frequency can no longer be determined. Interestingly, the difference between 1 m and 3 m is much more pronounced than the difference between 3 m and 5 m.

Results from the previous sections were obtained by using a camera lens with a field of view of 60 degrees. When a smaller FOV is used, the LED occupies a relatively larger portion of the image, and we may be able to detect it at greater distances. In order to test this hypothesis, we used a lens set (https://www.arducam.com/product/m12-mount-camera-lens-kit-arduino-raspberry-pi/, accessed on 12 March 2021) and varied the FOV from 10 to 60 degrees, at a fixed distance of 5 m. Again, the transmitter frequency was approximately 2 kHz, images were recorded during 30 s for every experiment, and the calibration procedure was used to estimate the transmitter frequency. Table 6 shows the accuracy of frequency estimation as a function of the FOV. It is clear that by sufficiently decreasing the FOV, the LED can still be detected. Even at a distance of 5 m, we can obtain the same accuracy of 95% as in Section 4.1 by using a lens with a FOV of 10 degrees. At shorter distances, a larger FOV can potentially also be used.

#### 4.2.5. Camera Resolution

**Figure 12:**
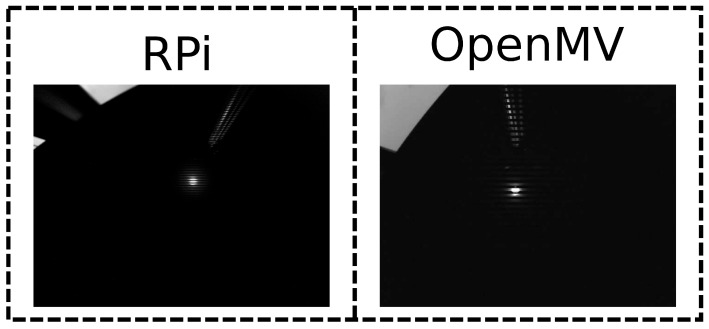
Example images of the same light taken with different cameras.

Similar to lighting conditions, camera resolution can have a significant impact on the performance of computer vision approaches. In the previous sections, the OpenMV M7 camera was used as an image sensor, which has a relatively low resolution of 640 × 480 pixels. We now hypothesize that a higher resolution will lead to a higher accuracy, for calibration of both the position and frequency of the LEDs. The increased resolution provides a higher granularity and thus potentially a greater accuracy in distinguishing the location of the light source. Additionally, a higher resolution provides additional stripes in the image, which may improve frequency detection. To verify this hypothesis, additional experiments were performed with a Raspberry Pi (RPi) camera sensor. Similar to Section 4.2.2 and Section 4.2.3, the robot followed a stop and go trajectory, and data were recorded for offline postprocessing. Figure 12 compares images captured with both cameras. The RPi camera is designed for the low-cost single-board computer of the same name, which is particularly popular for embedded applications. The maximum resolution of 3280 × 2484 pixels was used, in order to amplify any effects related to the resolution. Images taken at such a high resolution take up a lot of space in memory. Therefore, pictures were not continually recorded. Rather, 10 images were taken when the robot was located underneath the light source. Both types of cameras were equipped with a 60 degree FOV lens. Due to an error in data recording, there are only two experiments with the RPi camera instead of the usual three.

Table 7 contains the main quality metrics for calibration with both camera’s. The results appear to support our previous hypothesis. The accuracy of transmitter positioning is improved, although the improvement is rather small. A larger improvement can be seen in the frequency identification. The high-frequency source can now be identified much more accurately, due to the increased bandwidth of the RPi camera.

On the other hand, the time required to collect data with the RPi camera is more than double that of the OpenMV camera. The RPi camera is designed to be used with the Raspberry Pi single-board computer, which has significantly lower computational power compared to the laptop that collected the images from the OpenMV camera. Therefore, capturing each image takes a significantly longer amount of time. The main bottleneck here is the RPi itself, if one were to use a camera with a USB interface, a laptop could again be used to capture the images and calibration time would decrease. However, the duration of an experiment would likely still be higher with higher resolutions, yet less drastically so with the correct hardware. Processing time is also significantly increased when using the RPi camera. The higher resolution of the images means that the image processing takes a few seconds longer. The majority of the time increase can be attributed to the larger number of LIDAR measurements, which in turn is caused by the longer stationary time needed to capture the images with an RPi camera.

#### 4.2.6. Field of View

**Figure 13:**
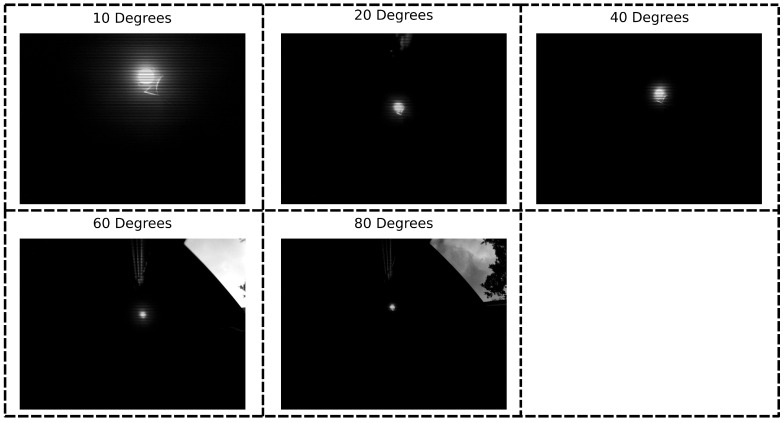
Example images for different FOV lenses.

Section 4.2.4 mentioned the effects of changing the field of view to improve the detection rate. Due to the nature of those experiments, we could not determine the error on the LED position. We therefore perform additional experiments with a changing FOV and multiple LEDs. These experiments were performed in the normal experimental setup (Figure 2). Section 4.2.5 showed that a larger resolution improves calibration performance. Therefore, we will again use this larger resolution camera in this section. Similar to Section 4.2.5, the RPi camera was used and images were only captured when the camera was approximately underneath a light source. By changing the lens, the FOV is again varied from 10 to 80 degrees. Figure 13 shows example pictures of the same light source for every FOV.

**Figure 14:**
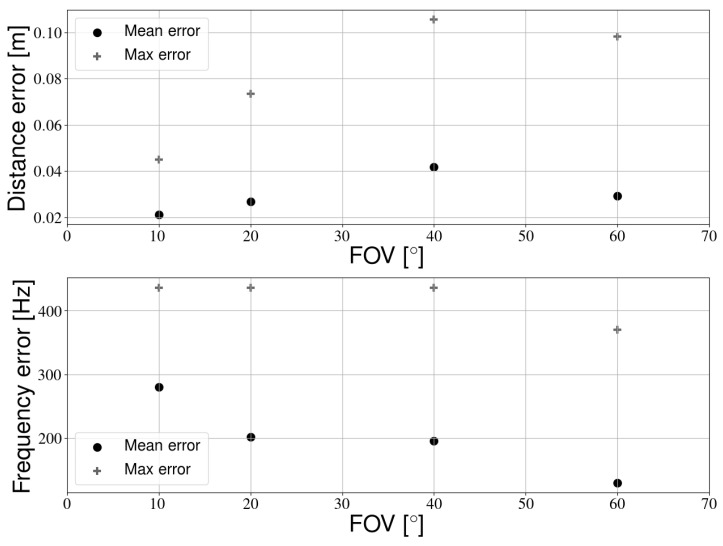
Top: positioning accuracy as a function of the camera FOV. Bottom: frequency accuracy as a function of the camera FOV.

Figure 14 shows the mean accuracy of transmitter positioning and frequency detection as a function of the FOV. From 80 degrees onward, the calibration procedure starts failing, resulting in large errors. For the sake of clarity, these results are not included in these figures. In general, a smaller FOV leads to a better positioning accuracy, though the improvement is relatively small. The exception to this is the 40 degree lens, which actually has a larger error compared to 60 degrees. In contrast, a smaller FOV actually lowers frequency detection accuracy. The 40 degree lens fits the overall trend better in this case. Therefore, one can trade off the positioning and frequency accuracy. However, the 60 degrees from previous sections seems to have already been a good compromise for our setup. As discussed in Section 4.2.4, a larger height may still necessitate a smaller FOV, as the light sources will become difficult to detect otherwise.

### 4.3. Influence on Positioning

The goal of a calibration procedure is to accurately measure the environmental parameters needed for determining the receiver location. Calibration errors will likely result in positioning errors, though this is often not a simple linear relation. Therefore, it is challenging to determine how accurately the calibration needs to be performed in order to guarantee adequate positioning performance in a later stage. In this section, we evaluate the impact of calibration errors on visible light positioning, in order to determine if our calibration provides satisfactory results.

First, we calibrated the setup with the configuration that was determined to be the best trade-off between position and frequency accuracy. More specifically, we used an RPi camera with a field of view of 60 degrees and drove the robot in a stop and go trajectory. This setup was also calibrated manually as a point of reference. In our previous work, we described sensor fusion based robot positioning with three filters, namely an Extended Kalman Filter (EKF), a Particle Filter (PF) and a hybrid Particle/Kalman filter (PaKa) [41]. We now use the parameters from both the manual and robot calibration in these positioning approaches. We used all data from the conditions described in Section 4.2.3. Positioning accuracy results were obtained in the same way as described in [41]. Contrary to previous sections, “positioning accuracy” does not refer to the accuracy on the position of the transmitters. In this section specifically, “positioning accuracy” refers to the accuracy on the robot position, the calculation of which is described in [41].

**Figure 15:**
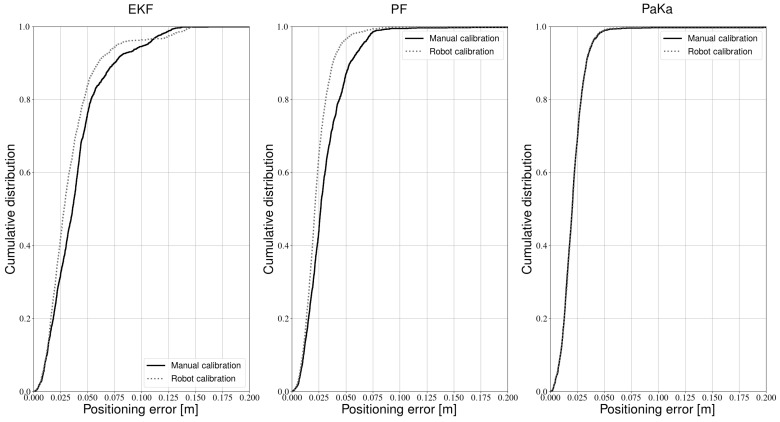
Cumulative positioning error distributions of calibration methods for different filters.

The results from all experiments were combined into one cumulative distribution per filter, which are shown in Figure 15. It is clear that the proposed calibration procedure has little impact on positioning accuracy. Occasionally, the new method even improves accuracy. However, as explained in [41], the accuracy results of the PF and the PaKa can have a small variation due to the sampling of probability distributions. In general, the robot calibration is more accurate, albeit only slightly. Of the three positioning approaches, the hybrid filter seems to be least impacted by the new calibration method. The difference between the error distributions of the PaKa filter in Figure 15 is often less than 1 mm. All filters also have no trouble identifying the lights correctly. Even though the new calibration method introduces an error on the modulation frequency, it is not large enough to cause ambiguity among the transmitters.

Note that this calibration was performed with an RPi camera but that positioning was performed with the OpenMV camera. The above results therefore show that device heterogeneity is not an issue that needs to be specifically taken into account, contrary to some RSS-based positioning systems [45,46,47,48].

## 5. Discussion

Amsters et al. [13] proposed a proof of concept for a calibration procedure of VLP systems. Contrary to [13], the improved procedure described in this paper does not require prior knowledge of the number of light sources. In the vast majority of cases, the algorithm was able to determine the correct number of light sources. When using the zigzag trajectory, or a FOV of 80 degrees, the number of transmitters could be underestimated. In all the other tests that were performed (29 experiments in total), the calibration algorithm correctly determined the number of LEDs.

This type of calibration procedure for VLP systems has not been used before. Therefore, it was unclear how robust the approach is and which factors can influence the results. During our parameter study (Section 4.2), we obtained several key insights. For example, a limitation of the procedure is that it cannot be used for calibrating transmitters modulated with sine waves, which is a consequence of using a camera as a receiver. However, the majority of VLP systems described in literature use On-Off Keying (OOK) as a modulation scheme, even if only frequencies are transmitted [49]. In case code division is used, researchers also often opt for OOK. While we performed experiments with FDMA as a multiple access technology, it would be relatively straightforward to include code division multiplexing by expanding the image processing pipeline. Another limitation is that we can only determine the two-dimensional position of the LEDs. Some positioning approaches require knowledge of the ceiling height, which would have to be measured separately.

The proposed calibration procedure was also not influenced significantly by the ambient lighting, similar to the positioning approach used as an evaluation [41]. In contrast, the robot trajectory, height, FOV and resolution all had an impact on calibration accuracy. A large resolution should be used to increase accuracy of both frequency detection and transmitter positioning. However, we recommend the use of a USB camera in order to capture pictures faster. In our experiments, a FOV of 60 degrees provided a good trade-off between positioning and frequency detection accuracy. In case the distance between transmitter and receiver is large (as is the case with high ceilings), a smaller FOV may be required to detect the light sources. Finally, care should be taken to stop the robot at each light source, rather than using a continuous motion. The latter could lead to poor accuracy and an underestimation of the number of light sources.

The main objective of the technique is to calibrate the parameters of the system, so that these can be used for positioning in a later stage. The experimental results in Section 4.3 showed that the parameters of the experimental setup can be determined with sufficient accuracy. The error on the light source locations did not result in increased positioning errors. In the case of our experimental setup, the transmitter frequencies could also be determined with sufficient accuracy so as to not cause ambiguity. It is important to note that one should take care that the modulation frequencies are sufficiently far apart, as some error is introduced when calibrating the modulation frequency. We should also note that certain positioning approaches are more susceptible to calibration errors than others. The positioning approach used as an evaluation tool made use of sensor fusion. In case of large measurement errors, the filters can fall back on odometry data. However, this is only the case when the error on the observation is sufficiently large. More subtle disturbances such as errors on the transmitter coordinates cannot be filtered. Additionally, with this work we showed that it is possible to close the loop between calibration and positioning. That is, we can efficiently calibrate the setup with a mobile robot and then use the determined parameters for high-accuracy positioning. Manual calibration also leads to errors on the transmitter locations. As evidenced by our positioning case, these errors are likely of the same order of magnitude as the robot calibration.

Our work shares similarities with robot-based RFID calibration. Hähnel et al. [11] also used a mobile robot equipped with a LIDAR and used it to reconstruct a map of the environment. The position of RFID tags was later estimated based on the path of the robot. Similarly, Milella et al. [12] also mapped indoor spaces with a mobile robot in order to localize RFID tags. They used fuzzy logic to determine the likelihood of a tag location. Mirowski et al. [16] proposed the use of a mobile robot for calibration of Wi-Fi localization systems. Contrary to [11,12], they used Quick Response (QR) codes to aid with loop closures, which raises the question as to how these QR codes should be localized.

Literature on the subject of VLP calibration is limited. Most examples focused on obtaining the parameters of the channel model [33,37], which we cannot calibrate. However, the approach which we used as an evaluation tool does not require these parameters as the channel model is not used [41]. This does limit the calibration procedure to mostly camera-based positioning systems. It is possible to further extend the proposed calibration system by including a photodiode on the robot platform and using the intensity measurements to obtain the parameters of the channel model.

In order to obtain the transmitter locations and identities, we obtained the receiver position through SLAM, rather than the manual measurements used in [31,50]. Contrary to [35], we were also able to obtain light source identities. Yue et al. [36] did use modulated LEDs, yet they have significantly lower accuracy compared to our work. However, our approach required a dedicated procedure rather than crowdsourcing the required data. Additionally, our robot needed to be manually driven by a human operator. Nevertheless, it may be possible to let the robot perform the calibration autonomously, whereas crowdsourcing will always require the cooperation of humans.

## 6. Conclusions

In this work, we outlined an improved calibration procedure for VLP systems, based on data collection with a mobile robot. The new approach had significantly improved performance compared to previous work. Accuracy of LED localization was almost doubled. Additionally, whereas previous work remained a proof-of-concept, we performed an extensive parameter study to characterize the strengths and limitations of the approach. Based on these results, we suggested the use of high resolution camera, with a FOV of 60 degrees to further improve the accuracy of LED placement and frequency detection. We showed that ambient lighting has little influence on the proposed procedure. Through positioning experiments, we determined that the approach is also accurate enough to calibrate high-performance VLP systems. In doing so, an important barrier to entry is removed for visible light positioning systems.

Our approach required a dedicated site survey, rather than crowdsourcing. While less convenient, it did result in much greater accuracy. The procedure was also unable to calibrate the channel model. In future work, we could add a photodiode to the sensor platform in order to obtain the Lambertian emission parameters. Additionally, we could investigate the possibility of letting the robot perform the procedure autonomously, in order to reduce the human labor required.

## Figures and Tables

**Table 1:** Calibration approaches for indoor positioning systems.

Reference	Positioning Technology	Calibration Method	Calibrated Parameters
**Calibration of fingerprinting systems**			
[9]	Wi-Fi	Robot site survey	RSS map
[10]	Wi-Fi	Robot site survey	RSS map
[15]	Wi-Fi	Robot site survey	RSS map
[16]	Wi-Fi/LTE	signalSLAM	RSS map
[17]	Magnetic field	signalSLAM	RSS map
[14]	Wi-Fi	signalSLAM	RSS map
[18]	Wi-Fi + PDR	crowdsourcing	RSS map
[19]	Wi-Fi	crowdsourcing	RSS map
[20]	Wi-Fi/Magnetic/PDR	crowdsourcing	RSS map
[21]	Wi-Fi	crowdsourcing	RSS map
**Calibration of range based systems**			
[7]	Ultrasound	Network optimization	Beacon coordinates
[8]	UWB	Autocalibration (initial solution), Followed by Network optimization	Range bias Beacon coordinates
[22]	Ultrasound	known locations	Beacon coordinates
[23]	Not specified	known locations	Beacon coordinates
[24]	UWB	known locations	Beacon coordinates
[25]	Wi-Fi	known locations	Beacon coordinates
[26]	UWB	Network optimization	Beacon coordinates
[27]	Ultrasound	Autocalibration Network optimization	Beacon coordinates
[28]	Ultrasound	known locations + Autocalibration	Beacon coordinates
[29]	Ultrasound	Network optimization	Beacon coordinates
**Calibration of VLP systems**			
[30]	VLP	known locations	Receiver parameters
[31]	VLP	known locations	Receiver parameters + Beacon coordinates
[32]	VLP	known locations	Receiver parameters
[33]	VLP	known locations	Channel model
[34]	VLP	known locations	Channel model
[35]	Wi-Fi/ambient light/magnetic field	signalSLAM	RSS map
[36]	VLP	signalSLAM	RSS map
[37]	VLP	known locations	Channel model
This work	VLP	Robot site survey	Beacon coordinates

**Table 2:** Hardware specifications.

Specifications	Value	Unit
**LED specifications**		
Frequency LED 1	1.57	kHz
Frequency LED 2	2.03	kHz
Frequency LED 3	2.87	kHz
Frequency LED 4	4.92	kHz
**OpenMV camera specification**		
Model	M7	
Resolution	640 × 480	pixels
Frame rate	30	fps
**RPI camera specification**		
Model	v2	
Resolution	3280 × 2484	pixels
Frame rate	≈0.75	fps
**LIDAR specifications**		
Model	RPLIDAR A1	
Measurement range	12	m
Measurement frequency	5.5	Hz
Angular resolution	1	°
Distance resolution	0.2	cm
**Robot specifications**		
Model	Kobuki	
Gyroscope measurement range	110	°/s
Odometry resolution	2578.33	tics/revolution
**Computer specifications**		
Model	Dell OptiPlex 5050	
CPU	3.6 × 4	GHz
RAM	16	GB

**Table 3:** Calibration parameters.

Parameter	Value	Unit
Minimum physical distance between lights	1	m
Minimum distance in frequency spectrum	0.1	kHz
Minimum number of occurrences	2	/
Maximum horizontal distance from image center (OpenMV)	100	pixels
Maximum horizontal distance from image center (RPi)	300	pixels

**Table 4:** Duration and processing time of calibration trajectories.

Trajectory	Average Duration [s]	Average Processing Time [s]
Zigzag	110.75	24.46
Stop and go	115.06	25.26

**Table 5:** Frequency estimation accuracy as a function of the transmitter–receiver distance. Field Of View (FOV) is kept fixed at 60 degrees.

Transmitter—Receiver Distance [m]	Detection Accuracy [%]
1.00	94.44
1.50	94.44
2.00	0.00
2.50	0.00
3.00	0.00
3.50	0.00
4.00	0.00
4.50	0.00
5.00	0.00

**Table 6:** Frequency estimation accuracy as a function of the FOV. Transmitter–receiver distance is kept fixed at 5 m.

FOV [°]	Detection Accuracy [%]
10	94.44
20	0.00
40	0.00
60	0.00

**Table 7:** Calibration results for different camera sensors.

Camera	OpenMV	RPI
Average position error [m]	0.06	0.04
Maximum position error [m]	0.13	0.11
Average frequency error [Hz]	236.66	124.51
Maximum frequency error [Hz]	820.00	370.09
Processing time [s]	25.26	63.18
Trajectory time [s]	115.06	265.94

## Data Availability

Not applicable.

## Appendix A

**Table A1:** Cost of the experimental setup.

Component	Amount	Cost Per Unit [€]
Mobile robot	1	603.79
LIDAR	1	119.79
LED	4	9.08
ITEM profiles	1	298.74
Lab bench power supply	1	296.45
Signal generator	4	109.09
OpenMV camera	1	76.17
RPi camera	1	26.98
RPi	1	42.2895
laptop	1	459
lens kit	1	82.89
Computer	1	1363.46
Total		3842.2395
